# Flipped classroom improves Omani nursing students performance and satisfaction in anatomy and physiology

**DOI:** 10.1186/s12912-020-00515-w

**Published:** 2021-01-02

**Authors:** Mickaël Antoine Joseph, Erna Judith Roach, Jansirani Natarajan, Suja Karkada, Arcalyd Rose Ramos Cayaban

**Affiliations:** 1 Fundamentals and Administration Department, College of Nursing, Sultan Qaboos University, Al Khoud, 123 Muscat, Oman; 2 Maternal and Child Health Department, College of Nursing, Sultan Qaboos University, Muscat, Oman

**Keywords:** Education, Nursing, Anatomy, Physiology, Active learning, Oman

## Abstract

**Background:**

Nursing students struggle with anatomy and physiology course because of the complicated terminology and the difficulty in handling large amounts of information. New, innovative instructional strategies must be integrated into nursing education to improve nursing students’ performance in this challenging bioscience course. The aim of this study was to determine the impact of an innovative teaching strategy, the flipped classroom, on the performance and satisfaction of Omani nursing students in an anatomy and physiology course.

**Methods:**

A quasi-experimental design was used with two classes of 112 first-year nursing students at the College of Nursing, Sultan Qaboos University, Oman. Online videos and active-learning activities about the respiratory system were developed and implemented in an anatomy and physiology course with 53 first-semester nursing students. The control group consisted of a previous cohort of 59 students enrolled in the same course but taught with a traditional lecture approach. The impact of the flipped classroom strategy was measured by students’ performance on the final examination and students’ self-reported satisfaction. Wilcoxon signed-rank and Mann-Whitney U tests were used to compare students’ academic performance.

**Results:**

Our results showed that the performance of the flipped classroom group was better than that of the traditional lecture group. The mean scores of students instructed with the flipped classroom method on the respiratory system items in the final examination were significantly higher than those of the control group, U = 1089.00, *z* = − 2.789, *p* < .005. Moreover, the results of a survey showed that nursing students were satisfied with the flipped classroom method. Overall, 68 to 78% of students agreed or strongly agreed that the flipped classroom method improved their learning and increased their interest in the course.

**Conclusion:**

Compared with the didactic lecture format, flipped classroom strategy improved Omani nursing students’ performance in and satisfaction with an anatomy and physiology course. These results show that the flipped classroom is an important teaching strategy in nursing education.

**Supplementary Information:**

The online version contains supplementary material available at 10.1186/s12912-020-00515-w.

## Background

At the beginning of the twenty-first century, researchers found a huge gap between nursing education and nursing practice, leading to calls for a fundamental transformation of nursing education [1, 2]. This call encouraged nursing teachers to plan learning experiences that will better prepare graduate nurses to practice in a rapidly changing health care environment. To that end, Benner [1] and MacKinnon et al. [2] proposed changes in the education system, such as moving away from teaching decontextualized knowledge, better integration of active learning in the classroom, and an increased emphasis on teaching clinical reasoning. New and innovative instructional strategies must be integrated within nursing education to achieve these goals.

Anatomy and Physiology (A&P) courses are crucial to nurses’ training [3], and correctly applying the knowledge learned in A&P to solve clinical problems is of paramount importance to nursing practice [4]. The inability of nurses to remember information from A&P and apply it to practice may have unfortunate consequences for patients and could be detrimental to the reputation of the nursing profession [5]. However, many nursing students have difficulties with the retention of large volumes of new vocabulary in anatomy and the complex physiological concepts involved. Studies have shown that many nursing students struggle to understand and remember A&P knowledge and have difficulty applying this content in clinical practice [6, 7]. Hence, nursing students perform poorly in this basic course, despite claiming to be very interested in the subject, and despite the efforts of nursing educators to engage them [5, 8].

The conventional approach to teaching A&P includes didactic lectures, guided exercises, and anatomy workbooks for the laboratory. This method of teaching is widely used; however, it has been shown to be less effective than several new and more interactive methods [9]. Notably, the flipped or inverted classroom has become popular as a teaching strategy in secondary education in the United States [10, 11]. This approach uses technology to combine didactic learning at home with interactive activities (e.g., discussion, exercises and clarification) in the classroom [12]. In other words, the nursing educator provides materials (e.g. pre-recorded multimedia lectures) that students can review outside of the classroom at their own pace, freeing up class time for other activities intended to consolidate students’ learning [13]. The out-of-classroom materials may include recorded PowerPoints, tutorial lectures in the form of videos, podcasts, notes, and animations [14]. This type of teaching centers students’ learning needs and offers them the opportunity to ask questions, clarify doubts and discuss ideas in the classroom, rather than passively sitting and listening [15].

Several researches have assessed the efficacy of the flipped classroom approach in undergraduate nursing students but the results were inconclusive [16]. While some studies found a beneficial effect of this approach on nursing students’ academic performance and satisfaction, others did not. For instance, Mikkelsen et al. (2015) flipped one chapter in an A&P course at a school of nursing in Denmark [17]. Students reported their opinion of the flipped classroom’s effect on their learning outcome in a survey. The results indicated that students found the strategy engaging and reported high satisfaction. However, this study did not measure differences in students’ performance and whether flipping the classroom improved students’ learning outcomes. In the United States, Missildine et al. used the flipped classroom teaching approach in an adult health course and found that it resulted in significantly better exam performance than traditional lecturing [12]. However, flipping the whole course resulted in reduced satisfaction among nursing students. These discrepancies in the level of satisfaction between studies have not yet been investigated. Moreover, all previous studies of the flipped classroom approach for nursing education have been conducted in the United States or Europe, where the language of instruction at the university level is the same as in secondary schools. Studies examining the efficiency of the flipped classroom approach in Oman have been scarce and limited mainly to English language teaching [18–20]. Therefore, the aim of this study was to determine the impact of flipping one A&P chapter on performance and satisfaction of Omani nursing students. This effect was measured in terms of students’ performance on the final examination and their self-reported level of satisfaction with the course.

## Methods

### Participants

The study was carried out with two classes of 112 first-year nursing students in the Fall 2017 and the Spring 2018 semester at College of Nursing, Sultan Qaboos University, Oman.

A quasi-experimental design was used in which a control group (CG) in fall 2017 semester (*N* = 59) was compared to an experimental group (EG) in spring 2018 semester (*N* = 53) to assess the efficiency of the flipped classroom instructional approach. Therefore, students in the CG were taught the respiratory system using a traditional lecturing method consisting of didactic PowerPoint presentations along with some activities such as multiple-choice questions (MCQs) and exercises to complete at home after class. Whereas students in the EG were exposed to a flipped classroom method where they had to watch recorded videos of the respiratory system at home before coming to class and class time was used for small work-group activities. Students from previous years indicated via course evaluations that learning the A&P of the respiratory system was challenging and therefore this subject was chosen to be taught using the flipped classroom instructional approach.

### Anatomy and physiology I course

All students were enrolled in a four-credit A&P I course, with five contact hours per week (3 h for lecturing and 2 h for a laboratory session). A&P I is a 15 week-long course taught in the first semester of the Bachelor of Nursing Program and includes the following chapters: introduction to anatomy, cytology, histology, skeletal system, muscular system, cardiovascular system, lymphatic system, respiratory system, and urinary system. The laboratory sessions consisted of a group-work in which students had access to plastic models to better understand the anatomical structures. The same assistant professor (first author) taught the course for both semesters (hence both groups CG and EG) using the same textbook and content.

### Flipped classroom method

While the CG was only subjected to traditional lecturing for all the aforementioned chapters, students in the EG were exposed to a flipped classroom method on one chapter only (respiratory system), whereas the other chapters were taught using the traditional way (didactic). One week before the class, the instructor recorded short videos (less than 10 min each using PowerPoint Office) covering the different objectives of the respiratory system lecture. Examples of the videos included anatomy of the nasal cavity, pharynx, larynx, trachea, bronchi, bronchioles, lungs, and physiology of breathing. These videos were uploaded to the learning management system Moodle and became available to the students in the EG. Students were instructed to watch the videos prior to the class. Along with the videos, captions were included to enhance students’ understanding. For the respiratory system, a total of 8 h’ class time was spread over 5 days (2 + 1 + 2//+ 2 + 1) for a period of 2 weeks for both groups. Of the 8 h, 2 h were dedicated to a laboratory session. For the EG, a lesson plan was posted on Moodle explaining the different elements of preparation that students needed to achieve before coming to the class. It was of paramount importance that students watched the assigned videos prior to class sessions. In order to ensure this, the instructor explained to the EG how the flipped classroom works and set expectations about the importance of pre-class preparation and the importance of watching the assigned videos before coming to class. Students were also warned that they would not be able to benefit from the exercises in class if they arrived unprepared. Moreover, an anonymous pre-quiz was carried out to assess students’ understanding of videos content. The instructor developed one quiz for each session (five quizzes in total) comprising of 10 MCQs each. These were simple questions that students should be able to answer if they had watched the videos and understood the content. These MCQs only emphasized the lowest level thinking skill (remembering) in Bloom’s taxonomy. The same anonymous quiz was given as a formative assessment at the beginning of the class session (pre-quiz) and the end of each session (post-quiz).

### Class activities

During the flipped classroom session, students were divided into six groups, and each group consisted of eight to nine students. The classroom was modified to accommodate small group seating around tables. Each session started with a fifteen-minute pre-quiz. Afterwards, students summarized the main points about concepts they learned in the videos (e.g. roles of the respiratory system, functions of the organs of the respiratory system, muscles of breathing, pressure gradients affecting the flow of air, process of gas exchange and transport of oxygen and carbon dioxide, etc.). Each group was given a set of exercises, case studies, and problems relevant to the video material they had watched (see Additional file 1). Each group was responsible for one objective and tasked to discuss about it for 15 min and solve the corresponding exercises. Then, the groups were mixed so students were able to solve all the exercises, discuss them with their peers, and learn together to achieve all the objectives. The instructor passed between the groups to listen to discussion, answer questions, solve discrepancies, and clarify concepts. Each session ended with a fifteen-minute post-quiz.

### Satisfaction survey

At the end of the last flipped classroom, students were asked to fill in an anonymous online questionnaire containing questions about their level of satisfaction about the flipped classroom method and the impact this approach had on their learning. This anonymous questionnaire was produced with Google Forms and made available to all students immediately after the last class session. Before starting the survey, students had an informed consent form to acknowledge explaining about the survey and the fact that it is not mandatory. The voluntary survey consisted of demographic questions (gender, age, grade point average, etc.) along with six items scored on a five-point Likert scale (strongly agree = 5, agree = 4, neither agree nor disagree = 3, disagree = 2, strongly disagree = 1). The survey also contained two open-ended questions about students’ perception of the main benefits and disadvantages of the flipped classroom in order to generate more in-depth qualitative data. The questionnaire focused on learning improvement, interest in and satisfaction with the course, improvement of grades, satisfaction with the flipped classroom and relevance of course material to the students’ careers. The survey had been used previously in a study on the application of the flipped classroom approach for nurses, and consent to use the survey was obtained from the original authors [15]. Cronbach’s alpha of the survey in our study was 0.930 indicating a reliable instrument.

### Performance

Both groups of students (EG and CG) were evaluated at the end of the semester with the same set of MCQs that was developed and used to assess knowledge and application of course content. Overall, 60 MCQs were developed and classified according to different learning levels of Bloom’s taxonomy [21] by two different authors. Following the College of Nursing regulations, the different MCQs were assigned to four cognitive-based classifications: remembering (30%), understanding (40%), applying (20%) and analysing (10%). Among the 60 MCQs, 16 exam questions were dedicated to the respiratory system (five remembering, five understanding, four applying, and two analysing items). Students were given 2 h to complete the final examination questions during week 16, which is a regularly scheduled finals week.

### Data analysis

All variables were tested with a Shapiro-Wilk test to assess for normal distribution. The result of the Shapiro-Wilk test was significant and therefore non-parametric tests were used. Pre-test and post-test scores of the quizzes were compared using the Wilcoxon signed-rank test. For the final examination, the difference between student’s performances on respiratory system questions was analysed using the Wilcoxon signed-rank test. The difference between students’ performance of the two cohorts was analysed using the Mann-Whitney U test. For the different quartiles, the respiratory system versus other items on the final examination were analysed using Wilcoxon signed-rank. To see whether there was a difference in the percentage of correct answers to different levels of Bloom’s taxonomy questions between the two cohorts, a Mann-Whitney U test was used. Internal consistency of the MCQs and the questionnaire was tested using Cronbach’s alpha. Wilcoxon signed-rank was also utilized to test whether the median scores for the questionnaire was higher than three (neither agree nor disagree) on a 5-point Likert scale. All statistical analyses were carried out using SPSS® version 23.

## Results

### Participants

Fifty-three students were enrolled in the A&P I course in the Spring of 2018 and were included in the EG. Of the 53 students, 49 completed the online survey. Of the 49 students, 83.7% were female, the average age was 18.84 years (standard deviation ±0.77), 30.6% had no grade point average as this was their first semester, and the majority of students (53.1%) had a normal course load of 12 to 13 credits. Overall, 40.8% of students reported that they had watched the assigned videos once before coming to class, 36.7% watched them twice, 18.4% thrice, 4.1% reported watching the videos five times or more and more importantly none of the students reported not having watched the videos prior to the class sessions (Table 1). Overall, 59 students were enrolled in the A&P I course in the Fall of 2017 (CG) out of which 71.2% were female and the mean age 18.29 years (standard deviation ±0.67). The demographic data of the participants was used to ensure homogeneity between the CG and the EG in terms of their general characteristics. No statistically significant differences were found in terms of gender (*p* = 0.261) and age (*p* = 0.07) between the CG and EG, indicating that the two groups were largely homogeneous.

**Table 1:** Demographics and background information of EG study participants and number of times students had watched the videos prior to classroom activities (*N* = 49). Note that 100% of students reported watching the videos at least once before class

Variables	Number of students (%)
Gender	
Females	41 (83.7%)
Males	8 (16.3%)
Age: Mean (standard deviation)	18.84 (0.77)
Cumulative grade point average	
No grade point average yet	15 (30.6%)
below 2.0	5 (10.2%)
2.01–2.49	9 (18.4%)
2.5–2.99	5 (10.2%)
3.0–3.49	10 (20.4%)
3.5 and above	5 (10.2%)
Number of credits for Spring 2018	
9 or below	2 (4.1%)
10–11	13 (26.5%)
12–13	26 (53.1%)
14–15	7 (14.3%)
16 and above	1 (2%)
Number of times students have watched the videos before class	
None (0)	0 (0%)
Once (1)	20 (40.8%)
Twice (2)	18 (36.7%)
Thrice (3)	9 (18.4%)
4 times	0 (0%)
5 times or more	2 (4.1%)

### Students’ performance

**Fig. 1:**
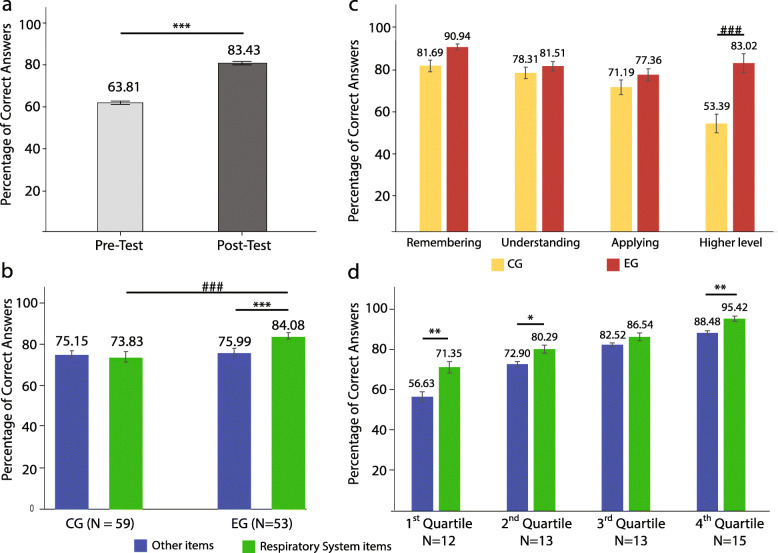
The impact of flipping the classroom on the percentage of correct answers in anatomy and physiology. **a** A comparison of students’ pre-test and post-test scores for the five quizzes administered for the EG (*N* = 53). Results are expressed as mean ± standard error of the mean (SEM) and converted into a percent class average. Before any in-class explanation or activity, students obtained an accuracy of 64% on the quizzes, showing that they had watched the videos prior to class. The active learning in class increased students’ performance by almost 20%. **b** Anatomy and physiology final examination average (in percent) for the EG in a flipped classroom (*N =* 53) was significantly higher than the average of the CG in a traditional didactic lecture (*N* = 59). Results are expressed as percentage of correct responses mean ± SEM on the respiratory system items compared to the other items. Improvement in scores was 8% better with flipped classroom than with didactic lecturing. Bars graphs are plotted separately by group for CG and EG. Statistically significant differences are indicated by *** Wilcoxon signed-rank test *p* < 0.001 and ### Mann-Whitney U test *p* < 0.001. **c** Students in a flipped classroom course (EG) performed significantly better than the previous cohort (CG) on high-order analysis multiple-choice questions in the final examination. Statistically significant differences are indicated by ### Mann-Whitney U test, *p <* 0.001. **d** Difference in students’ semester average grades between respiratory items (flipped classroom) and other items (traditional lecture) in an anatomy and physiology course for EG (*N* = 53). Students were divided into quartiles based on their final score on the final examination with 38–68% (1st quartile, *N =* 12), 69–80% (2nd quartile, *N* = 13), 81–85% (3rd quartile, *N* = 13), and 86–100% (4th quartile, *N* = 15). Each stratified group scored higher on the respiratory system items compared to the other items. The Wilcoxon signed-rank test was used for comparisons and significant *p* values are indicated. Error bars represent SEM; * < 0.05; ** < 0.01

We evaluated students’ knowledge before and after each flipped classroom session. The results show that students performed significantly better on the post-test compared to the pre-test (83.43 ± 16.65 versus 63.81 ± 22.02, Z = -11.752, *p* < 0.0001). However, even before any in-class activity, students scored almost 64% on the MCQ quizzes, which is above chance level, showing that students had watched the videos and understood the content prior to class. The scores on all five quizzes are presented as a mean ± standard error of the mean (SEM) in Fig. 1a and tested with Wilcoxon signed-rank for significance because the distribution of data was not normal.

To determine the effectiveness of the A&P flipped classroom method on students’ performance, the final examination grade of both groups (CG and EG) was examined. A total of 60 MCQs were utilized in the final examination, with 16 items dedicated to the respiratory system. The point biserial index for item discrimination of the exam questions were 0.39 ± 0.16 and 0.25 ± 0.15 respectively. The flipped classroom approach significantly improved students’ performance on the MCQs when compared to traditional lecture in the same group (EG) and with the previous group (CG). At the final examination, 3 weeks after the flipped classroom sessions, the performance was higher on the respiratory system (taught with the flipped classroom) than on the non-respiratory system questions (taught by the traditional method) for the EG students (84.08 ± 11.54% versus 75.99 ± 13.28%, *N* = 53, Z = -5.102 *p* < 0.0001). Moreover, in comparison to the CG of the previous semester, EG students’ grades were significantly higher on the respiratory system items. EG students’ performance was higher by 10.3% compared to the CG (84.08 ± 11.54% versus 73.83 ± 18.91%, *N =* 53 versus *N =* 59, Mann Whitney U = 1089; *p* = 0.005) (Fig. 1b).

Respiratory system questions were tallied based on the four cognitive based classifications of remembering, understanding, applying, and analysing. The percentages of correct answers of the two groups (CG and EG) were compared. The flipped classroom approach significantly enhanced students’ performance on the high-order analysis MCQs compared to performances on the traditional method (83.02 ± 33.89% versus 53.39 ± 35.80 Mann Whitney U = 852.5; *p* < 0.0001) (Fig. 1c).

In order to assess whether the benefits of the flipped classroom varied between the different student levels, the EG was divided into quartiles based on their score on the final MCQs examination. The median was 81% on a 100% examination score. The four groups were 38–68% (1st quartile, *N* = 12), 69–80% (2nd quartile, *N* = 13), 81–85% (3rd quartile, *N =* 13), and 86–100% (4th quartile, *N* = 15). In order to evaluate significance, a Wilcoxon signed-rank test was carried out on the differences of performance on the respiratory system and other items. Each quartile group had a higher percentage of correct answers on the respiratory system items after a flipped classroom than on the other items taught with lectures. However, only in the third quartile statistically significant differences were not observed (Fig. 1d).

### Students’ satisfaction

**Fig. 2:**
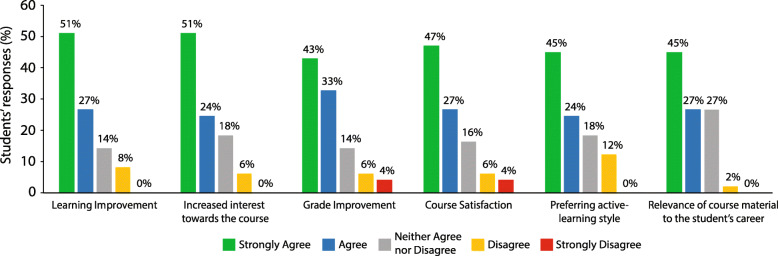
Students’ responses to the use of the flipped classroom approach. Students agreed or strongly agreed that flipped classroom improved their learning, increased their interest in the course, improved their grades, is more satisfying, is better when using active-learning style, and the course material in the flipped classroom is relevant to their career (78, 75, 76, 74, 69, and 72% of students respectively) than didactic lecturing. Results are presented as mean percentage (*N* = 49)

Following the last flipped classroom session, a brief online questionnaire was administered to determine students’ level of satisfaction with the flipped classroom compared to their satisfaction with the didactic lectures. The questionnaire was filled out anonymously, and the results are presented in Fig. 2. The questionnaire results revealed that 68 to 78% of students in the EG agreed or strongly agreed on the efficiency of the flipped classroom on improving their learning and their interests in the course. Compared with the didactic lecture format, students perceived the flipped classroom method to be more satisfying, interesting and capable of improving their grade and learning (Wilcoxon signed-rank, *p* < 0.0001 for the six questions compared with the median score of three for neither agree nor disagree). However, a small percentage of students (2 to 12%) disagreed or strongly disagreed with the statements and the use of the flipped classroom method.

In the open-ended section of the questionnaire, students mentioned that the flipped classroom instructional approach allowed them to “be more focused in class,” “access the explanation at their convenience,” “better remember the information,” and “understand clearly the respiratory system.” On the other hand, a large number of students commented that the flipped classroom method is very time-consuming. The other challenge of the approach, as expressed by the students, was mainly technical (weak Wi-Fi).

## Discussion

Studies of flipped classrooms in higher education are scarce, and only a limited number have focused on the effectiveness of this approach in nursing education [17, 22–24]. To the best of our knowledge, this study is the first to test the effectiveness of flipping the classroom when teaching Arabic-speaking nursing students.

The main finding of this study is that Omani nursing students can learn basic A&P more effectively using a flipped classroom approach than by means of didactic lectures. The use of online videos prior to class combined with active learning in class resulted in better performance than the traditional approach.

These results suggest that students in the flipped classroom (EG) did benefit from the respiratory system lecture taught with the flipped classroom approach. This is aligned with the findings of other studies that have addressed the effect of online teaching outside the classroom and in-class active-learning methods, which showed significant improvement in nursing students’ performances compared to a control group [15, 25]. Flipping only one chapter in the semester allowed this study to hold some variables constant. It allowed comparison among the same group (respiratory system items versus other systems in the EG) and between different groups (respiratory system items in the EG compared with the same respiratory system items in the CG). Direct comparison was possible because the final examinations for both the EG and CG were identical.

A flipped classroom approach is not effective if students do not come prepared (having watched the assigned videos prior to class). These videos constitute the building blocks on which students will rely for the whole class session. All class meetings for the EG in this study started with group exercises about the material viewed, which ensured that students applied the acquired knowledge. None of the students in this study reported not having watched the videos prior to class. This could be due in part to the relatively short length of the videos; several studies have emphasized the importance of keeping educational videos short [26–29], and that longer educational videos can result in lower student satisfaction [30]. Quizzes were administered at the beginning and end of each session ensured that students benefited most from the class exercises and activities. Studies have used quizzes as primary resources to assess students’ understanding of the videos watched at home and found that they increase learners’ motivation and enhance the flipped classroom experience [31].

Incorporation of small-group activities is vital, and so is finding the correct group structure [32]. It is difficult to have a homogenous group of students working together when the class is flipped for the whole semester. One of the main strengths of this study is that the instructor knew the students very well, and assigned groups based on students’ level of understanding. Each group incorporated both weak and strong students, with the latter acting as peer instructors to those who required more assistance. The peer instructors also benefited from this approach, as the act of explaining the material to their classmates may have consolidated their knowledge of the subject. The small group activities in class allowed students to ask questions they could not or would not have asked during a traditional lecture, and to interact more with their peers and teacher. More than 75% of students preferred flipped instruction to the traditional method and expressed their satisfaction with it, results that echo the findings of other studies [15, 17]. One of the main motivations behind changing from traditional lectures to online videos to be watched prior to class was to free up more class time for active learning. Previous studies have reported that decreasing face-to-face class time when online videos were used and found that the student performance was unaffected [33]. However, we suggest keeping the amount of face-to-face class time unchanged and adding an online component to be viewed prior to class to achieve better learning outcomes.

Nursing students must develop the critical thinking skills necessary to solve patients’ problems and foster decision-making skills for nursing interventions [34]. It has been shown that higher-level questions (analysis) can foster in-depth learning and help students in subsequent courses [35, 36]. In our study, questions were divided into four categories according to Bloom’s taxonomy: remembering, understanding, applying and analysing questions [37]. Students in the flipped classroom group performed better in all learning domains than students taught with traditional lectures, though the data showed statistical significance only in the higher (analysis) learning category. These results should encourage teachers to utilize in-class activities and use online content to foster higher-level analysis and critical thinking skills [38].

Based on marks in the final examination, the flipped classroom approach resulted in improved performance by students in all quartiles. The biggest increase was seen in lower-quartile academic achievers. This method also reduced the achievement gap between low and high achievers by almost 8%. These findings are in line with those of Gross and colleagues [39], who showed that the flipped classroom had the greatest effect on the performance of low achievers in a biochemistry course. Another study showed a long-term effect of flipped classroom on the performance of low achievers after testing students in a subsequent course [40]; in that study, the flipped classroom approach benefited low achievers in a gross anatomy course to a greater degree than their higher-achieving peers, and this improved performance was sustained in a subsequent kinesiology course. Thus, our study adds to the previous evidence that the flipped classroom benefits low achievers more than high achievers and reduces the achievement gap.

Omani nursing students’ perception of and satisfaction with the flipped classroom approach was investigated. A survey was used to obtain more insight into students’ interest in the course, improvement of grades, and level of satisfaction with the flipped approach and the active-learning style. Responses to the online survey showed that around 75% of the students found the flipped-classroom approach more satisfying than traditional lectures. This reflects the findings of a study by Mikkelsen’s exploring nursing students’ experiences, perceptions and behaviour during an A&P course in which one chapter was taught using a flipped classroom design [17]. That study found that 80% of nursing students were very satisfied with this new teaching method. However, most studies in the literature have reported high levels of dissatisfaction with the use of flipped classroom in nursing education. For instance, Misseldine and colleagues demonstrated that nursing students taught in a flipped classroom were less satisfied than students taught via other methods [12]. Similar results were observed by Post and colleagues, who found that nursing students were frustrated by and expressed discomfort and anxiety with the flipped classroom approach [38]. In both our study and those in the literature, students reported that the flipped classroom approach required more work. However, in our study, only around 25% of students had a negative perception of the flipped classroom approach. This discrepancy in results could be explained by the fact that both Mikkelsen’s and our study flipped material from only one chapter of the A&P course, while the other chapters were taught via traditional methods. Misseldine and colleagues flipped the classroom for the entire semester, which may have led students to be less satisfied.

Although we found that nursing students were satisfied with flipping one chapter in an A&P course, we wonder how many chapters can be flipped before students’ perceptions and level of satisfaction changes. Is there a threshold that should not be exceeded? Would flipping four chapters prove less satisfactory than the one chapter we tested? Indeed, is there an optimal amount of material to be flipped that will produce the best benefit to learners? Further research into the reasons behind these differences in satisfaction with the flipped classroom is necessary.

### Limitations

This study had several limitations. First, only one eight-hour portion of a 75 h-course was flipped. We believe that this was not sufficient to fully foster higher analysis and critical thinking skills in nursing students. The personalized instruction of the flipped classroom should continue throughout the semester in order to identify and assist weak students [37]. The flipped classroom allows the teacher to be more approachable and available to answer students’ questions and address misconceptions. However, we believe that more than 2 weeks is needed to ensure that students fully benefit from this approach.

Further, only short-term knowledge was assessed when lectures were moved to online videos. The flipped classroom approach uses active learning methods, which could help enhance students’ long-term memory. Additional studies should investigate the impact of this instructional approach over the longer term. Moreover, quizzes were completed anonymously, so we were not able to recognize students who have not carefully watched the video recordings prior to class. Therefore, it was not possible to correlate the quiz results with the final examination and determine whether insufficient study of the videos affected students’ performance.

Finally, the survey was not comprehensive and was limited to only six items and two open-ended questions. The use of a short survey was intended make it brief, easy and convenient to answer, and led to a 92.5% response rate. Although the survey had open-ended questions, the answers did not provide a comprehensive overview of which parts of the flipped classroom approach (online videos, in-class active learning, quizzes) caused the reported high level of satisfaction and the observed effects on performance. Collection of such information could be valuable when flipping the classroom in the future.

## Conclusions

We have shown that flipping the classroom can improve Omani nursing students’ performance in and satisfaction with introductory A&P. The flipping of instruction for one chapter in an A&P course resulted in improved final examination grades and enabled students to apply their analytical skills. This new educational approach had a greater effect on the performance of low achievers than on high achievers and reduced the achievement gap between low- and high-achieving students. Furthermore, survey responses suggest that students prefer the flipped classroom to the traditional lecture-based method of teaching. Thus, this study shows the benefits of flipping only a portion of a bioscience course in improving nursing students’ academic performance and satisfaction. Notably, it is the first to show the beneficial effect of this instructional approach among native Arabic speakers studying A&P in English.

## Supplementary Information

**Additional file 1.**

## Data Availability

The datasets generated and/or analysed during the current study are not publicly available to preserve anonymity of the respondents but are available from the corresponding author on reasonable request. The authors declare that they have no competing interests.

## Abbreviations

A&P: Anatomy and physiology
CG: Control group
EG: Experimental group
MCQs: Multiple choice questions
SEM: Standard error of the mean
