# Supplementary material for: Flipped classroom improves Omani nursing students performance and satisfaction in anatomy and physiology
Source: BMC Nurs. 2021 Jan 2;20:1. doi: 10.1186/s12912-020-00515-w (PMC7778794; doi:10.1186/s12912-020-00515-w)
Supplement: Supplementary file 1 — Additional file 1. [file 12912_2020_515_MOESM1_ESM.pdf]

<u>**Respiratory System Exercises (Part 1)**</u>

The upper respiratory system warms, humidifies, and filters air

This figure illustrates the skeletal and cartilaginous framework of the nose.

Identify the various bones and cartilages contributing to that framework

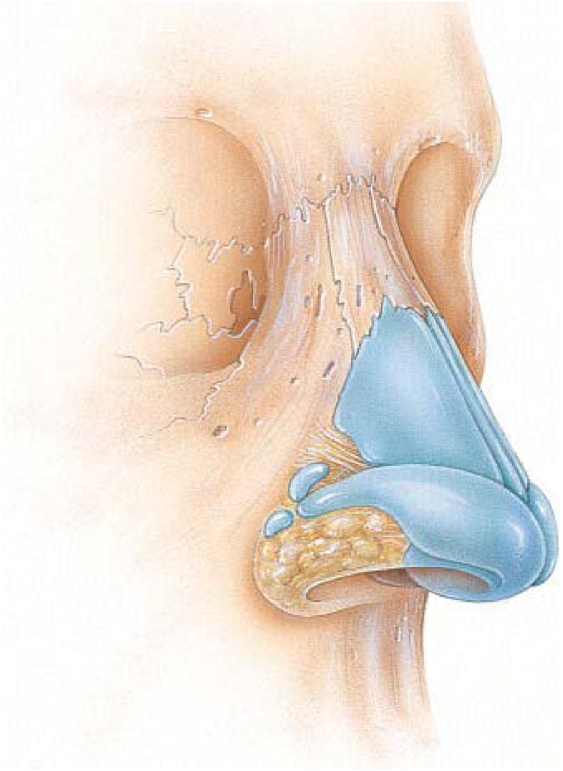

1. Alar cartilage
2. Septal cartilage
3. Lateral nasal cartilage
4. Maxillary bone
5. Nasal bone
6. Frontal bone

Complete the following statements concerning nasal cavity and adjacent structures

Air enters the nasal cavities of the respiratory system through the _______________. The nasal cavity is divided by the midline _______________. The nasal cavity mucosa has several functions. Its major functions are to _____________, _____________________, ________________ the incoming air. Mucous-membrane-lined cavities called ________________ are found in several bones surrounding the nasal cavities. They make the skull less heavy and probably act as resonance chambers for ______________. The passageway common to the digestive and respiratory systems, the _____________________, is often referred to as the throat, it connects the nasal cavities with the _______________ below. Clusters of lymphoid tissue, _________________, are part of the defensive system of the body.

Respiratory System Exercises (Part 3)

Exercise 1: Highlight the correct words or phrases in each of the following sentences.

1. Air moves in and out of lungs because of a (pressure)(oxygen) gradient.

2. When the pressure within the lungs drops lower than atmospheric pressure, (inspiration)(expiration) occurs; when it rises above atmospheric pressure, (inspiration)(expiration) occurs.

3. The thin film of fluid between the visceral and parietal pleura (causes them to cling together) (allow them to glide painlessly against each other), aiding lung expansion.

4. The potential space between the two pleura maintains a pressure slightly (less)(greater) than atmospheric pressure; this is called (negative)(positive) pressure.

Exercise 2: Match each air pressure with the statements that apply to each. Use each letter once. Each answer line will have two correct letters.

1) Atmospheric pressure ________________	A. The pressure in the bronchial tree and alveoli
2) Intrapleural pressure__________________	B. the pressure of the air around us
3) Intrapulmonic (alveolar) pressure _______	C. The pressure within the potential pleural space
	D. 760 mmHg at sea level
	E. Fluctuates below and above atmospheric pressure during breathing
	F. Always slightly below atmospheric pressure

Exercise 3: Label each of the following statements as True (T) or False (F).

1. Inhalation is the active phase of quiet breathing ________
2. During quiet breathing, exhalation does not require any muscle contraction ______
3. The diaphragm rises when it contracts _______
4. The size of the thoracic cavity decreases during exhalation ___________
5. Atmospheric pressure is less than lung pressure during inhalation _________
6. The diaphragm relaxes during exhalation _________
7. During inhalation, changes in lung volume causes air to enter the lungs ________

Exercise 4: Add the word or words that correctly complete each of the following statements.

1. The underlying principle of breathing is that air flows from the region of high pressure to a region of ______________________.

2. Pressure changes occurring in the lungs can be traced to the activity of skeletal muscles known as ______________________.

3. During inspiration, the ribs are raised upward and outward by a set of respiratory muscles called _____________________.

4. During inspiration, contractions cause the downward movement of a dome-shaped muscle known as the __________________.

5. The relaxation of respiratory muscles compresses the thorax and increases the air pressure in the ____________________.

6. While inspiration is an active process, the process of expiration is ________________.

Exercise 5: Write the appropriate term in each blank.

1. The substance in the fluid lining the alveoli that prevents their collapse

   ____

2. The ease with which the lungs and thorax can be expanded ________________
3. Air flows most easily when the airway diameter is ________________

Exercise 6: Fill in the blanks to complete the sentences about measuring lung capacities.

1. The amount of air inhaled and exhaled during quiet breathing is known as ___________. In a healthy young adult, this measurement is typically ________ mL.

2. The amount of air inhaled using maximum effort after a normal inspiration is called the __________________ volume. This amount is typically _______________ mL.

3. The amount of air that can be exhaled after a normal expiration using maximum effort is the _____________________ volume. This amount is typically ____________ mL.

4. The amount of air remaining in the lungs after a forced expiration is called the ________________. This amount is about _____________ mL.

5. The amount of air that can be inhaled and exhaled with the deepest possible breath is the **vital capacity**. (Vital capacity is the tidal volume combined with the inspiratory and expiratory reserve volumes)
6. **Total lung capacity** is the maximum amount of air that the lungs can contain: the vital capacity plus the residual volume.

Example of a pre-test post-test Multiple Choice Question

1. Which one of the following muscles is the **secondary** muscle of inhalation?

   A. Diaphragm
   B. External intercostals
   C. Internal intercostals
   D. Sternocleidomastoid

2. Which one of the following muscles is **NOT** engaged in forceful inhalation?

   A. Sternocleidomastoid
   B. Internal intercostals
   C. Scalene
   D. Pectoralis minor

3. Which of the following muscles is engaged in forceful exhalation?

   A. Sternocleidomastoid
   B. Abdominal muscles
   C. Internal intercostals
   D. Diaphragm

4. According to Boyle's law of gases, if the size of a container is decreased to half, the pressure of the gas inside the container will ______.

   A. decrease to half
   B. double
   C. triple
   D. decrease

5. What do we call the amount of air that enters and exists the lungs during quiet breathing?

   A. Tidal volume
   B. Minute ventilation
   C. Inspiratory reserve volume
   D. Expiratory reserve volume

6. If the tidal volume is 500 ml/breath and the respiratory rate is 12 breaths/min. How much will the minute ventilation be?

    A. 5 liters/min
    B. 6 liters/min
    C. 7 liters/min
    D. 8 liters/min

7. What do we call the conducting airways that does not undergo respiratory exchange?

    A. Tidal volume
    B. Minute ventilation
    C. Anatomic dead space
    D. Alveolar ventilation rate

8. If the tidal volume is 500 mL and the anatomic dead space is 200 mL. How much air actually reaches the respiratory zone?

    A. 500 mL
    B. 200 mL
    C. 300 mL
    D. 700 mL

9. If the amount of air that reaches the respiratory zone is 350 mL/breath and the respiratory rate is 12 breaths/min. How much is the alveolar ventilation rate?

    A. 6 liters/min
    B. 5.2 liters/min
    C. 4.2 liters/min
    D. 3 liters/min

10. The diaphragm descends, and the rib cage expands due to contraction of the external intercostal muscles. This movement results in a larger thoracic cavity, which decreases the pressure within the lungs so that it is lower than the surrounding atmospheric pressure (2 mmHg less than 760). This will cause:
    A. Air to move from outside to the lungs
    B. Air to move from the lungs to the outside
